# *Babesia microti-*induced fulminant sepsis in an immunocompromised host: A case report and the case-specific literature review

**DOI:** 10.1515/biol-2022-0448

**Published:** 2022-09-14

**Authors:** Harry A. Conte, Michael C. Biondi, Sok-Ja Janket, Leland K. Ackerson, Eleftherios P. Diamandis

**Affiliations:** Department of Infectious Diseases, Saint Francis Hospital, Hartford, CT, USA; Department of Infectious Diseases, Johnson Memorial Hospital, Stafford Springs, CT, USA; Department of Radiology, Saint Francis Hospital, Hartford, CT, USA; Center for Clinical and Translational Research, The Forsyth Institute, Cambridge, MA, USA; Department of Public Health, University of Massachusetts at Lowell, Lowell, MA, USA; Department of Pathology and Laboratory Medicine, Mount Sinai Hospital, 60 Murray St. Box 32, Floor 6, Rm L6-201. Toronto, ON, M5T 3L9, Canada

**Keywords:** *Babesia microti*, intra-erythrocytic parasite, eukaryotic pathogens, chronic lymphocytic leukemia, recrudescence

## Abstract

*Babesia microti* is an obligate intra-erythrocytic parasite transmitted by infected ticks. *B. microti* is a eukaryote much larger than prokaryotic microbes and more similar to human hosts in their biochemistry and metabolism. Moreover, *Babesia* spp. possess various immune evasion mechanisms leading to persistent and sometimes life-threatening diseases in immunocompromised hosts. Chronic lymphocytic leukemia (CLL) is the most prevalent adult B-cell malignancy, and a small percentage of CLL transforms into aggressive lymphomas. CLL also causes immune dysfunction due to the over-expansion of immature and ineffective B-cells. When our patient with indolent CLL presented with anemia, pancytopenia, and splenomegaly, all his healthcare providers presumptively assumed a malignant transformation of CLL. However, these are also the signs and symptoms of babesiosis. Herein, we report a case where *B. microti* infection was presumed as a malignant transformation of CLL and narrowly avoided a devastating outcome. Although the patient developed fulminant sepsis, he finally received the correct diagnosis and treatment. Unfortunately, the disease recrudesced twice. Each time, it became more difficult to control the infection. We describe the clinical course of the case and discuss the case-specific literature review. This report highlights the importance of differential diagnoses ruling out infections which include babesiosis, prior to initiating the treatment of B-cell malignancy.

## 1 Introduction

Human babesiosis is a zoonotic infection caused by *Babesia* spp. transmitted by Ixodid ticks [1]. *Babesia* spp. are obligate intraerythrocytic parasites belonging to the phylum *Apicomplexa,* which have the unique ability to penetrate and lyse human erythrocytes [2]. Single-cell eukaryotes are five to seven orders of magnitude larger than average prokaryotes [3] and are biochemically, metabolically, and genetically more similar to their human hosts than prokaryotes. For these reasons, once they infect a host, they are difficult to eradicate without harming the host [4,5]. They also have sophisticated organelles and various immune evasion mechanisms leading to persistent and sometimes life-threatening diseases in immunocompromised hosts [6,7]. Another reason for their persistence is that they develop resistance to the drugs used to treat the disease [4]. Also, gene mutations in the parasite can cause treatment failure [8,9]. We have illustrated the location of these eukaryotic protists in the evolutionary ladder (Figure S1).

Pathogenesis requires several vectors and steps which have been described in numerous previous reviews [10,11]. Once inside the human body, the motile spore-like sporozoites invade the erythrocytes using the apical complex. *Babesia* multiplies inside erythrocytes asexually, with each *Babesia* budding into two to four daughter cells (merozoites) and continuing to infect adjacent erythrocytes using their gliding ability [7]. Eventually, infected erythrocytes lyse and cause symptoms such as hemolytic anemia, hemoglobinuria, and jaundice [12]. These symptoms are often misdiagnosed as hematologic malignancies [13,14].

Symptoms of babesiosis range from asymptomatic (about 20% of the cases) or mild, while others may develop a severe or even fatal course of disease depending on the hosts’ immune status [15]. Although early babesiosis symptoms are non-specific, with fever and malaise, severe cases may present dyspnea, splenomegaly, hepatomegaly (or both), anemia, jaundice, hemoglobinuria, hypotension, leukopenia, and thrombocytopenia [12]. Of note, splenomegaly, hemolytic anemia, thrombocytopenia, and many of its physical symptoms are similar to those of stage 4 chronic lymphocytic leukemia (CLL). Thus, it is important to differentiate babesiosis from the advanced stages of CLL.

CLL is the most prevalent adult B-cell malignancy, representing 30% of all adult leukemias [16]. The progression of CLL is heterogeneous. In some patients, it remains indolent through their life expectancy, while in others, it can exacerbate acute aggressive forms easily reaching stages 3 or 4 in Rai’s staging system [17]. The signs and symptoms of advanced CLL are very similar to those of babesiosis. These include malaise, fever, anemia, leukopenia, thrombocytopenia, splenomegaly, and/or pain in the upper left abdomen due to splenomegaly [18]. Although babesiosis and advanced CLL present similar signs and symptoms, these two diseases require diametrically different treatments [19]. CLL requires immune-suppressive anti-neoplastic therapy, while babesiosis requires immune-enhancing anti-infective treatment. Thus, it is of utmost importance to differentiate babesiosis from advanced CLL.

**Figure 1:**
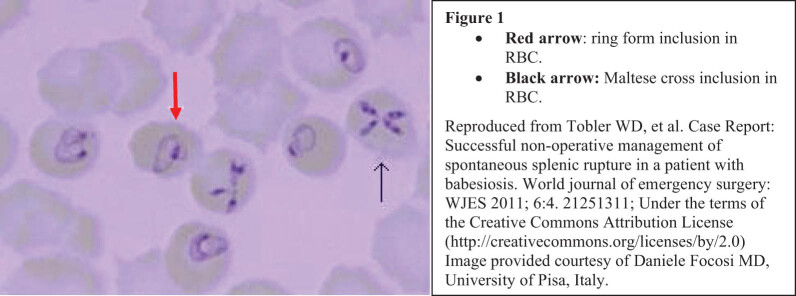
Ring forms and Maltese cross inclusions in erythrocytes.

History of tick bite or residing in or traveling to endemic areas may give clues for potential risks of babesiosis, but a more specific diagnosis of babesiosis is necessary. When parasitemia is high, the diagnosis will be made by peripheral blood smears [12] demonstrating common intra-erythrocytic ring forms of the parasite (red arrow, Figure 1) or rarely by a pathognomonic tetrad configuration of merozoites resembling a Maltese cross [10] (black arrow, Figure 1). The diagnosis can be confirmed by reverse transcriptase polymerase chain reaction (rt-PCR), targeting the 18 S rRNA gene of *Babesia microti* from whole blood, as described in the reference [20].

### 1.1 Epidemiology of babesiosis

The most predominant species identified in the eastern and midwestern United States is *B. microti,* while *B. divergens* is common in Europe. In recent years, *B. duncani* was identified in the western United States [21,22]. More than 100 *Babesia* spp. have been identified, and different geographic regions and varied animal host different *Babesia* species [1]. These geography-specific *Babesia* species were reported in detail in previous publications [10,11].

The most common mode of infection is through the bites of infected ticks, but often patients do not recall the incidences of tick bites [12]. Less commonly, babesiosis can be transmitted through blood transfusions. Although the incidence is low, transfusion-transmitted babesiosis is much more dangerous because these patients commonly have serious comorbidities requiring transfusion. Very rarely an infected mother can transmit babesia to the newborn [12].

The incidence of babesiosis has been steadily increasing in the United States. The cumulative incidence of babesiosis in the United States between 2011 and 2015 was 7,612 cases (6,277 confirmed and 1,335 probable) [23]. Of these, 7,194 cases (94.5%) occurred in seven states: Connecticut, Massachusetts, Minnesota, New Jersey, New York, Rhode Island, and Wisconsin [23]. The risk factors for severe babesiosis are extremely young or old age; immunosuppression due to malignancy, organ transplantation, or splenectomy; and persons with chronic heart, liver, or kidney diseases [11]. Our patient is old, has heart disease, has malignancy, and resides in one of the seven states of the United States where babesiosis is endemic.

Between 1983 and 1994 in Wisconsin, three fatalities occurred (30%) among ten reported cases. All three deaths occurred in patients who were immunocompromised by asplenia or receiving high-dose steroids [24]. Thus, among the immunocompromised hosts, babesiosis should be recognized as a serious health threat [23].

### 1.2 Clinical course of the case

In late October 2019, a 76-year-old male with a history of indolent CLL and heart failure (HF) presented at a cardiology annual checkup. The cardiologist noticed anemia, leukopenia, and thrombocytopenia. Suspecting a malignant transformation of CLL, the cardiologist recommended an oncology consult. Patient’s leukocyte count was 2,000/µL (reference range: 4,000–10,500/µL), erythrocyte count was 3 × 10^6^/µL (reference range: 4.7–6.0 × 10^6/^µL), and platelet count was 43 × 10^3^/µL (reference range: 150–450 × 10^3^/µL). He had lost about 6.2 kg in the previous month. He had never smoked, never used any recreational drugs, and did not drink any alcohol.

His past medical history was notable with paroxysmal ventricular fibrillation leading to bundle branch block and heart failure in 2007, which was treated with medical management and Implantable Cardioverter Defibrillator (ICD) implantation. These interventions resulted in an excellent outcome with no physical limitation (NYHA category 1). During the heart failure diagnosis, CLL was identified incidentally but did not require any treatment for the past 15 years. His lymphocyte count fluctuated between 23,000 and 50,000/µL (normal range: 720–4,100/µL) without any other symptoms.

**Figure 2:**
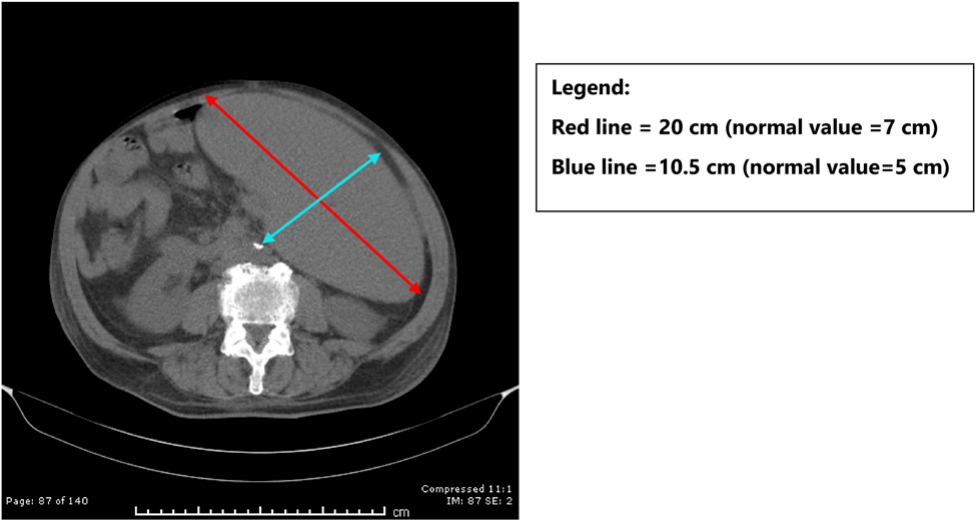
CT scan showing massive splenomegaly.

In early November 2019, the patient sought oncology consult where a computerized axial tomography (CT) scan revealed massive splenomegaly with dimensions of 22 cm × 10.5 cm × 20 cm, (normal spleen size: 12 cm × 5 cm × 7 cm; Figure 2), mediastinal lymphadenopathy, but no significant atherosclerotic changes or cardiomegaly were observed. However, serum immunoglobulins, albumin, and total protein were all low. Hypogammaglobulinemia is one of the criteria for malignant transformation of CLL [25]. His oncologist’s diagnosis was a malignant transformation to lymphoma, and he recommended immunotherapy with rituximab and ibrutinib. This is particularly important because advanced CLL is usually treated with corticosteroids, rituximab, or ibrutinib. With occult babesiosis in the background, these treatments could have been deadly as has been reported previously [24].

Serendipitously, the patient was aware of the serious adverse effects associated with the drugs of choice for CLL, ibrutinib [26,27,28] and rituximab [29,30,31], and he opted for an antioxidant resveratrol supplementation instead. Resveratrol induces apoptosis of malignant lymphocytes *in vitro* [32] and *in vivo* [33] Antioxidant supplementation started at the beginning of January 2020, generating one week of symptomatic relief, but by the second week, the patient’s symptoms had progressively worsened with severe pallor, dyspnea, and muscle weakness. For the next 3 weeks, the patient was too weak to get out of bed.

In the middle of February 2020, the patient developed shaking chills, acute respiratory distress syndrome, and delirium. He was transferred to the emergency department (ED) at the local hospital. At the ED, his temperature was 38·7°C, BP was 80/43 mmHg, pulse 124 beats per minute, breathing 20 breaths per minute, and sequential organ failure assessment (SOFA) score was 8. The SOFA score ≥8 is associated with a 21% hospital mortality rate [34].

ED physician’s diagnosis was sepsis. His laboratory findings at the ED are presented in Table 1. Immediately, supportive care to manage sepsis was initiated, including fluid resuscitation, controlling fever, and supplementing oxygen. Simultaneously, several diagnostic tests to determine the cause of sepsis were undertaken. The tests for pneumonia, pancreatitis, pyelonephritis, hepatitis, and acute decompensated heart failure were carried out, and none were significant. CT scan without contrast showed the same massive splenomegaly as was observed at the oncology visit. Urinalysis and urine culture were negative, and the ICD and its wires were in a good position. The ED physician noted unusual intracellular inclusions in the RBCs of the peripheral blood smear and consulted the infection specialist. After examining the specimen, the infection specialist (HC) determined that the inclusion bodies were the ring-form intraerythrocytic parasites (Figure 1) and made a diagnosis of babesiosis. The attending pathologist concurred with the diagnosis. Additionally, real-time PCR with an FDA-approved and clinically validated assay (Quest Diagnostics) confirmed the diagnosis by the presence of *B. microti* DNA in the blood. Tests for *Ehrlichia muris-like, Ehrlichia ewingii/canis, Ehrlichia chaffeensis, Anaplasma phagocytophilum, Streptococcus pneumoniae, Legionella,* and Lyme antibody were all negative.

**Table 1:** Laboratory test results of the patient at the emergency department

	Patient’s value	Reference range
WBC (per μL)	900	4,000–10,500
Differential (count)		
Neutrophils	500	1,800–7,800
Lymphocytes	300	1,000–3,200
RBC (per μL)	2.54 × 10^6^	4.7–6.0 × 10^6^
Hemoglobin (g/dL)	7.7	13.5–18.0
Hematocrit (%)	24.8	40–54
Platelets (per μL)	21 × 10^3^	150–450 × 10^3^
MPV (fL)	13.2	7.4–11.4
Amylase (U/L)	32	29–103
Lipase (U/L)	31	11–82
BNP (pg/mL)	23	0–100
Troponin (ng/mL)	0.03	<0.04
Lactate (mmol/L)	1.3	0.5–2.2
Total bilirubin (mg/dL)	2.3	0.3–1.0
Bilirubin, direct (mg/dL)	0.5	0.0–0.2
AST (SGOT) (U/L)	35	5–40
ALT (SGPT) (U/L)	24	7–52
LDH (U/L)	235	125–220
Alkaline phosphatase (U/L)	78	34–104
Smear interpretation	Rare intracellular inclusion in RBC observed	Consulted infectious disease specialist
Urea (mg/dL)	65	9–20
Creatinine (mg/dL)	1.7	0.7–1.3
Glomerular filtration rate (mL/min)	39.4	>60.0
Calcium (mg/dL)	7.4	8.4–10.2

fL, Femtoliter (10^−15^); BNP, B-type natriuretic peptide.

Once the diagnosis was made, aggressive anti-parasitic and antibiotic treatments were initiated by establishing a central venous catheter. The patient received a standard regimen for babesiosis treatment, a 10-day course of atovaquone 750 mg b.i.d. and concurrent azithromycin 500 mg q12 h. Also, he received an RBC-replacement transfusion. The patient was well-recovered and discharged after 4 days in the hospital. He completed the 10-day course of atovaquone and azithromycin treatment at home. The patient was able to do all his normal physical activities for the next 2 months.

Unfortunately, babesiosis recurred twice after the first discharge from the hospital, and the patient was re-admitted with a second severe sepsis. Although the patient is still taking the medications after 14 months after the second admission, he can do most of his physical activities. The recrudescent part of babesiosis will be detailed in our next report.


**Informed consent:** Informed consent has been obtained from all individuals included in this study.
**Ethical approval:** The research related to human use has been complied with all the relevant national regulations, institutional policies and in accordance with the tenets of the Helsinki Declaration, and has been approved by the author’s institutional review board or equivalent committee.

## 2 Discussion

This case clearly illustrates that eukaryotic infections such as babesiosis can become life-threatening illnesses in immunocompromised patients. It should be noted that his comorbid CLL involves B-cell defects resulting in impaired immunity, which increases the threat of severe babesiosis and recrudescence. Furthermore, babesiosis and advanced CLL symptoms are extremely similar, both presenting anemia, pancytopenia, and splenomegaly. Therefore, careful differential diagnosis is crucial.

The postulated causes for recrudescence are *Babesia* spp. express variant erythrocyte surface antigen1, which facilitates their adhesion to other cells [29] and forms a cluster with other RBCs [35]. These clusters can resist the hydrodynamic force of the bloodstream [35] and escape the splenic elimination of *Babesia* spp. [36,37]. These RBC clusters localize in the microvasculature [35], and *Babesia* spp. may avoid detection using PCR test. However, *B. microti-*specific clusters were not observed in the previous autopsy study [38]. We are not certain whether this autopsy study examined microvasculature. Future studies inspecting microvasculature are warranted.

Additionally, PCR has excellent sensitivity (96.2%) but poor to moderate specificity (70.5%), which carries approximately 30% false negative rates [39]. Although the negative PCR tests indicate the absence of detectable babesia DNA in the blood of approximately 70% (specificity) of the tested, the other 30% could have parasitemia that is below the detection levels of PCR [39]. This patient had negative PCR tests continuously since the first discharge. Nevertheless, babesiosis recrudesced twice. A marker of true negativity of babesia infection is desperately needed. One group used haptoglobin levels as a marker for the true negativity of babesia infection [9]. This test is based on the principle that highly oxidative-free hemoglobin originating from RBC lysis forms a haptoglobin–hemoglobin (Hp–Hb) complex, thereby avoiding oxidative tissue damage [40]. Hp–Hb complex lowers the serum haptoglobin levels, and the complex is eliminated by CD163-mediated endocytosis by macrophages through the spleen or other organs in the reticuloendothelial system [41]. This patient’s haptoglobin level was below the lowest detectable level for nearly 2 years. Additionally, the differential diagnosis should consider a history of Lyme disease, malaria, acute anemia, Colorado tick fever, Ehrlichiosis, typhoid fever, and CLL.

### 2.1 Differentiating CLL and babesiosis

As we described in the introduction, advanced CLL presents many symptoms of severe babesiosis and differential diagnosis becomes difficult when two pathologies coincide. Furthermore, the treatment modalities are diametrically different [17]. According to the 2018 International Workshop on CLL (IWCLL), guidelines recommend starting CLL therapy [42] if *any one of the following criteria* is satisfied: (a) hemoglobin <10 g/dL or platelet count of <100 × 10^9^/L; (b) massive (≥6 cm below the left costal margin) splenomegaly; (c) symptomatic splenomegaly; (d) presence of disease-related symptoms such as unintentional weight loss of ≥10% within the previous 6 months, or significant fatigue [42]. This patient’s initial hemoglobin level was 9·7 g/dL, platelet count was 45 × 10^9^/L, and he had massive splenomegaly. Thus, this patient satisfied the IWCLL criterion for CLL treatment. IWCLL criterion for treatment appears too non-specific and overly lenient. This patient could have received CLL treatment which might have generated disastrous consequences.

### 2.2 Hemophagocytic lymphohistiocytosis (HLH)

In immunocompetent patients, several cases of HLH following *B. microti* infection have been reported [43,44]. HLH is an aggressive and life-threatening syndrome of excessive immune activation. HLH can be categorized as primary and secondary. Primary HLH is a genetics-derived pathology usually occurring in children [45]. Secondary HLH is a rare but potentially lethal complication following severe infections or malignancies with high mortality [46]. B-cell malignancies, including CLL, are less prone to develop HLH [47,48] compared with the patients who have T- or natural killer cell-associated lymphoma [47].

### 2.3 Autoimmune hemolytic anemia (AIHA) following babesiosis

Babesiosis can cause non-immune hemolytic anemia due to the lysis of RBC by the parasite, as well as hemolytic anemia via autoimmunity [49]. AIHA is defined as the “destruction of RBCs through autoimmune mechanisms mediated by autoantibodies against erythrocyte surface antigens” [50]. Several studies reported post babesiosis (PB)-related AIHA, which occurred predominantly in asplenic patients [51,52]. Narurkar et al. stated that post-babesia AIHA is different from other types of AIHA because non-PB-related AIHAs are usually treated with splenectomy, but PB-associated AIHAs occur among asplenic patients [53]. Because PB-associated AIHAs occurred all in asplenic patients, [49] and asplenia increases autoimmune reactions, [54,55] asplenia may be the driver of AIHA. In pathological stress conditions such as asplenia, the liver takes over the removal of effete RBCs with monocytes acting as transient macrophages [56]. Further research on PB-associated AIHA is needed to elucidate whether post-babesiosis immune activation induces autoimmune reaction or asplenia triggers autoimmune hemolysis to remove senescent erythrocytes.

### 2.4 Cancer immunotherapy and eukaryotic infections

Ibrutinib and rituximab are the drugs of choice for the treatment of CLL. These drugs revolutionized CLL treatment with less toxicity than chemotherapy, but they suppress inflammatory pathways, which impair immune defense and can induce eukaryotic infections [57]. The patients who received Rituximab experienced relapses and persistent babesia infection [29]. Ibrutinib, which irreversibly inhibits Bruton’s tyrosine kinase, induces immune dysfunction and can cause serious fungal infections [27,58]. Immune suppression due to heavy corticosteroid usage has increased the risk of eukaryotic infections, Mucormycosis, after COVID-19 infection [59].

### 2.5 Resveratrol and immune suppression

It is highly likely that the resveratrol our patient took might have blunted the immune responses to babesiosis and contributed to severe sepsis. The innate immune system expresses TNF-α, IL-6, and IL-1β to defend the host from invading pathogens. Resveratrol suppresses NF-kB, TNF-α, and MAPK (mitogen-activated protein kinase) [27,58,60,61], and suppresses inflammatory responses against pathogens [62,63]. Resveratrol supplementation has resulted in mortalities among multiple myeloma patients in phase II clinical trial [64], and antioxidants supplementation was associated with poor survival among cancer patients [65].

## 3 Conclusions

When CLL patients present with anemia, pancytopenia, and splenomegaly, a careful differential diagnosis should be made to rule out the infectious origin of symptoms such as babesiosis before proceeding to anti-CLL treatment. First, a blood smear should rule out the presence of babesia parasites in the blood and then confirm the results with rt-PCR. We hope our patient’s journey will offer an opportunity for a better understanding of the complexities of eukaryotic infections such as babesiosis and the difficulties in treating them in immunocompromised hosts.

## Supplementary Material

Supplementary Figure

## References

[1] Mylonakis E. When to suspect and how to monitor babesiosis. Am Family Phys. 2001;63(10):1969–74. 11388711

[2] Jacot D, Tosetti N, Pires I, Stock J, Graindorge A, Hung YF, et al. An apicomplexan actin-binding protein serves as a connector and lipid sensor to coordinate motility and invasion. Cell Host Microbe. 2016;20(6):731–43. 10.1016/j.chom.2016.10.020. 27978434

[3] Lynch M. The origins of eukaryotic gene structure. Mol Biol Evol. 2006;23(2):450–68. 10.1093/molbev/msj050. Epub 2005 Nov 9. 16280547

[4] Fairlamb AH, Gow NA, Matthews KR, Waters AP. Drug resistance in eukaryotic microorganisms. Nat Microbiol. 2016;1(7):16092. 10.1038/nmicrobiol.2016.92. PMC5215055 27572976

[5] Janket S-J, Conte HA, Diamandis EP. Do Prevotella copri and Blastocystis promote euglycaemia? Lancet Microbe. 2021;2(11):e565–6. 10.1016/S2666-5247(21)00215-9 35544079

[6] Morrissette NS, Sibley LD. Cytoskeleton of apicomplexan parasites. Microbiol Mol Biol Rev. 2002;66(1):21–38. 10.1128/MMBR.66.1.21-38.2002. PMC120781 11875126

[7] Frénal K, Dubremetz JF, Lebrun M, Soldati-Favre D. Gliding motility powers invasion and egress in Apicomplexa. Nat Rev Microbiol. 2017;15(11):645–60. 10.1038/nrmicro.2017.86. 28867819

[8] Capela R, Moreira R, Lopes F. An overview of drug resistance in protozoal diseases. Int J Mol Sci. 2019;20(22):5748. 10.3390/ijms20225748. PMC6888673 31731801

[9] Simon MS, Westblade LF, Dziedziech A, Visone JE, Furman RR, Jenkins SG, et al. Clinical and molecular evidence of atovaquone and azithromycin resistance in relapsed babesia microti infection associated with rituximab and chronic lymphocytic leukemia. Clin Infect Dis: An Off Publ Infect Diseases Soc Am. 2017;65(7):1222–5. 10.1093/cid/cix477 PMC6248624 28541469

[10] Krause PJ. Human babesiosis. Int J Parasitol. 2019;49(2):165–74. 10.1016/j.ijpara.2018.11.007 30690090

[11] Vannier E, Krause PJ. Human babesiosis. N Engl J Med. 2012;366(25):2397–407. 10.1056/NEJMra1202018 22716978

[12] CDC. Parasite – Babesiosis Atlanta. Georgia: HHS; 2018. https://www.cdc.gov/parasites/babesiosis/disease.html.

[13] Akel T, Mobarakai N. Hematologic manifestations of babesiosis. Ann Clin Microbiol Antimicrob. 2017;16(1):6. 10.1186/s12941-017-0179-z PMC5310009 28202022

[14] Bläckberg J, Lazarevic VL, Hunfeld KP, Persson KEM. Low-virulent Babesia venatorum infection masquerading as hemophagocytic syndrome. Ann Hematol. 2018;97(4):731–3. 10.1007/s00277-017-3220-6 29285582

[15] Vannier E, Krause PJ. Update on babesiosis. Interdiscip Perspect Infect Dis. 2009;2009:984568. 10.1155/2009/984568 Epub 2009 Aug 27. PMC2734943 19727410

[16] Knittel G, Liedgens P, Reinhardt HC. Targeting ATM-deficient CLL through interference with DNA repair pathways. Front Genet. 2015;6:207. 10.3389/fgene.2015.00207 PMC4461826 26113859

[17] Rozovski U, Hazan-Halevy I, Keating MJ, Estrov Z. Personalized medicine in CLL: Current status and future perspectives. Cancer Lett. 2014;352(1):4–14. 10.1016/j.canlet.2013.07.013 PMC3871981 23879961

[18] Hallek M. Chronic lymphocytic leukemia: 2017 update on diagnosis, risk stratification, and treatment. Am J Hematol. 2017;92(9):946–65. 10.1002/ajh.24826 28782884

[19] Bucktrout SL, Bluestone JA, Ramsdell F. Recent advances in immunotherapies: From infection and autoimmunity, to cancer, and back again. Genome Med. 2018;10(1):79. 10.1186/s13073-018-0588-4 PMC6208073 30376867

[20] Krause PJ, Telford S 3rd, Spielman A, Ryan R, Magera J, Rajan TV, et al. Comparison of PCR with blood smear and inoculation of small animals for diagnosis of Babesia microti parasitemia. J Clin Microbiol. 1996;34(11):2791–4. 10.1128/jcm.34.11.2791-4.1996. PMC229405 8897184

[21] Herwaldt BL, Linden JV, Bosserman E, Young C, Olkowska D, Wilson M. Transfusion-associated babesiosis in the United States: A description of cases. Ann Intern Med. 2011;155(8):509–19. 10.7326/0003-4819-155-8-201110180-00362 21893613

[22] Karkoska K, Louie J, Appiah-Kubi AO, Wolfe L, Rubin L, Rajan S, et al. Transfusion-transmitted babesiosis leading to severe hemolysis in two patients with sickle cell anemia. Pediatr Blood Cancer. 2018;65(1). 10.1002/pbc.26734. Epub 2017 Aug 2. 28766838

[23] Gray EB, Herwaldt BL. Babesiosis surveillance – united states, 2011-2015. Morbidity Mortal Wkly Rep Surveill Summaries (Washington, DC: 2002). 2019;68(6):1–11. 10.15585/mmwr.ss6806a1 31145719

[24] Herwaldt BL, Springs FE, Roberts PP, Eberhard ML, Case K, Persing DH, et al. Babesiosis in Wisconsin: A potentially fatal disease. Am J Tropical Med Hyg. 1995;53(2):146–51. 10.4269/ajtmh.1995.53.146 7677215

[25] Pangalis GA, Angelopoulou MK, Vassilakopoulos TP, Siakantaris MP, Kittas C. B-chronic lymphocytic leukemia, small lymphocytic lymphoma, and lymphoplasmacytic lymphoma, including Waldenström’s macroglobulinemia: A clinical, morphologic, and biologic spectrum of similar disorders. Sem Hematol. 1999;36(2):104–14. 10319379

[26] Mato AR, Nabhan C, Thompson MC, Lamanna N, Brander DM, Hill B, et al. Toxicities and outcomes of 616 ibrutinib-treated patients in the United States: A real-world analysis. Haematologica. 2018;103(5):874–9. 10.3324/haematol.2017.182907 PMC5927982 29419429

[27] Chamilos G, Lionakis MS, Kontoyiannis DP. Call for action: Invasive fungal infections associated with ibrutinib and other small molecule kinase inhibitors targeting immune signaling pathways. Clin Infect Dis. 2018;66(1):140–8. 10.1093/cid/cix687 PMC5850040 29029010

[28] Salem JE, Manouchehri A, Bretagne M, Lebrun-Vignes B, Groarke JD, Johnson DB, et al. Cardiovascular toxicities associated with ibrutinib. J Am Coll Cardiol. 2019;74(13):1667–78. 10.016/j.jacc.2019.07.056. 31558250

[29] Krause PJ, Gewurz BE, Hill D, Marty FM, Vannier E, Foppa IM, et al. Persistent and relapsing babesiosis in immunocompromised patients. Clin Infect Dis. 2008;46(3):370–6. 10.1086/525852. 18181735

[30] Norris LB, Georgantopoulos P, Rao GA, Haddock KS, Bennett CL, Dorn WJB. Association between rituximab use and progressive multifocal leukoencephalopathy among non-HIV, non-Hodgkin lymphoma Veteran’s Administration patients. ASCO Annu Meet J Clin Oncol. 2014;e19540.

[31] Roberts DM, Jones RB, Smith RM, Alberici F, Kumaratne DS, Burns S, et al. Rituximab-associated hypogammaglobulinemia: Incidence, predictors and outcomes in patients with multi-system autoimmune disease. J Autoimmunity. 2015;57:60–5. 10.1016/j.jaut.2014.11.009 25556904

[32] Billard C, Izard JC, Roman V, Kern C, Mathiot C, Mentz F, et al. Comparative antiproliferative and apoptotic effects of resveratrol, epsilon-viniferin and vine-shots derived polyphenols (vineatrols) on chronic B lymphocytic leukemia cells and normal human lymphocytes. Leuk Lymphoma. 2002;43(10):1991–2002. 10.1080/1042819021000015952. 12481898

[33] Tomic J, McCaw L, Li Y, Hough MR, Ben-David Y, Moffat J, et al. Resveratrol has anti-leukemic activity associated with decreased O-GlcNAcylated proteins. Exp Hematol. 2013;41(8):675–86. 10.1016/j.exphem.2013.04.004. Epub Apr 15. 23597602

[34] Jones AE, Trzeciak S, Kline JA. The sequential organ failure assessment score for predicting outcome in patients with severe sepsis and evidence of hypoperfusion at the time of emergency department presentation. Crit Care Med. 2009;37(5):1649–54. 10.1097/CCM.0b013e31819def97 PMC2703722 19325482

[35] Schetters T. Mechanisms involved in the persistence of babesia canis infection in dogs. Pathog (Basel, Switz). 2019;8(3):94. 10.3390/pathogens8030094 PMC6789894 31261942

[36] Allred DR. Babesiosis: Persistence in the face of adversity. Trends Parasitol. 2003;19(2):51–5. 10.1016/s1471-4922(02)00065-x 12586467

[37] Krause PJ, Daily J, Telford SR, Vannier E, Lantos P, Spielman A. Shared features in the pathobiology of babesiosis and malaria. Trends Parasitol. 2007;23(12):605–10. 10.1016/j.pt.2007.09.005 17988944

[38] Clark IA, Budd AC, Hsue G, Haymore BR, Joyce AJ, Thorner R, et al. Absence of erythrocyte sequestration in a case of babesiosis in a splenectomized human patient. Malar J. 2006;5:69. 10.1186/475-2875-5-69. PMC1552079 16887045

[39] Akoolo L, Schlachter S, Khan R, Alter L, Rojtman AD, Gedroic K, et al. A novel quantitative PCR detects Babesia infection in patients not identified by currently available non-nucleic acid amplification tests. BMC Microbiol. 2017;17(1):16. 10.1186/s12866-017-0929-2. PMC5237571 28088177

[40] Nantasenamat C, Prachayasittikul V, Bulow L. Molecular modeling of the human hemoglobin-haptoglobin complex sheds light on the protective mechanisms of haptoglobin. PLoS One. 2013;8(4):e62996. 10.1371/journal.pone.0062996 PMC3637213 23638175

[41] Schaer DJ, Vinchi F, Ingoglia G, Tolosano E, Buehler PW. Haptoglobin, hemopexin, and related defense pathways-basic science, clinical perspectives, and drug development. Front Physiol. 2014;5:415. 10.3389/fphys.2014.00415 PMC4211382 25389409

[42] Hallek M, Cheson BD, Catovsky D, Caligaris-Cappio F, Dighiero G, Döhner H, et al. iwCLL guidelines for diagnosis, indications for treatment, response assessment, and supportive management of CLL. Blood. 2018;131(25):2745–60. 10.1182/blood-2017-09-806398 29540348

[43] Go SA, Phuoc VH, Eichenberg SE, Temesgen Z, Beckman TJ. Babesia microti infection and hemophagocytic lymphohistiocytosis in an immunocompetent patient. Int J Infect Dis. 2017;65:72–4. 10.1016/j.ijid.2017.09.026 28993284

[44] Kennedy-Snodgrass C, Obayomi M, Muddasani R, Slonim LB, Braunstein M. Hemophagocytic lymphohistiocytosis secondary to Babesia in an immunocompetent adult. Am J Hematol. 2019;94(3):379–83. 10.1002/ajh.25364 30478854

[45] Henter JI, Horne A, Aricó M, Egeler RM, Filipovich AH, Imashuku S, et al. HLH-2004: Diagnostic and therapeutic guidelines for hemophagocytic lymphohistiocytosis. Pediatric Blood Cancer. 2007;48(2):124–31. 10.1002/pbc.21039 16937360

[46] Fisman DN. Hemophagocytic syndromes and infection. Emerg Infect Dis. 2000;6(6):601–8. 10.3201/eid0606.000608 PMC2640913 11076718

[47] Han AR, Lee HR, Park BB, Hwang IG, Park S, Lee SC, et al. Lymphoma-associated hemophagocytic syndrome: Clinical features and treatment outcome. Ann Hematol. 2007;86(7):493–8. 10.1007/s00277-007-0278-6. Epub 2007 Mar 9. 17347847

[48] Tiong IS, Lau MB, Toumoua S, Chiruka S. A case of hemophagocytic lymphohistiocytosis in a patient with chronic lymphocytic leukemia after treatment with fludarabine, cyclophosphamide, and rituximab chemotherapy, with autopsy findings. Case Rep Hematol. 2012;2012:326053. 10.1155/2012/326053 Epub 2012 Dec 17. PMC3600305 23533846

[49] Rajapakse P, Bakirhan K. Autoimmune hemolytic anemia associated with human babesiosis. J Hematol. 2021;10(2):41–5. 10.14740/jh820 Epub 2021 Apr 27. PMC8110228 34007364

[50] Berentsen S, Barcellini W. Autoimmune hemolytic anemias. N Engl J Med. 2021;385(15):1407–19. 10.056/NEJMra2033982 34614331

[51] Santos MA, Tierney LM Jr, Manesh R. Babesiosis-associated warm autoimmune hemolytic anemia. J Gen Intern Med. 2020;35(3):928–9. 10.1007/s11606-019-05506-5 PMC7080882 31713032

[52] Woolley AE, Montgomery MW, Savage WJ, Achebe MO, Dunford K, Villeda S, et al. Post-babesiosis warm autoimmune hemolytic anemia. N Engl J Med. 2017;376(10):939–46. 10.1056/NEJMoa1612165 28273010

[53] Narurkar R, Mamorska-Dyga A, Nelson JC, Liu D. Autoimmune hemolytic anemia associated with babesiosis. Biomarker Res. 2017;5:14. 10.1186/s40364-017-0095-6 PMC5385069 28405337

[54] Di Sabatino A, Rosado MM, Cazzola P, Riboni R, Biagi F, Carsetti R, et al. Splenic hypofunction and the spectrum of autoimmune and malignant complications in celiac disease. Clin Gastroenterol Hepatol. 2006;4(2):179–86. 10.1016/s1542-3565(05)00982-1 16469678

[55] Giuffrida P, Aronico N, Rosselli M, Lenti MV, Cococcia S, Roccarina D, et al. Defective spleen function in autoimmune gastrointestinal disorders. Intern Emerg Med. 2020;15(2):225–9. 10.1007/s11739-019-02129-w 31214883

[56] Theurl I, Hilgendorf I, Nairz M, Tymoszuk P, Haschka D, Asshoff M, et al. On-demand erythrocyte disposal and iron recycling requires transient macrophages in the liver. Nat Med. 2016;22(8):945–51. 10.1038/nm.4146 PMC4957133 27428900

[57] Reynolds KL, Sullivan RJ, Fintelmann FJ, Mansour MK, England J. Case 9-2020: A 64-Year-Old man with shortness of breath, cough, and hypoxemia. N Engl J Med. 2020;382(12):1150–9. 10.1056/NEJMcpc1909621 32187473

[58] Ghez D, Calleja A, Protin C, Baron M, Ledoux MP, Damaj G, et al. Early-onset invasive aspergillosis and other fungal infections in patients treated with ibrutinib. Blood. 2018;131(17):1955–9. 10.1182/blood-2017-11-818286 29437588

[59] Stone N, Gupta N, Schwartz I. Mucormycosis: time to address this deadly fungal infection. Lancet Microbe. 2021;2(8):e343–e4. 10.1016/S2666-5247(21)00148-8 35544192

[60] Ruchlemer R, Ben-Ami R, Bar-Meir M, Brown JR, Malphettes M, Mous R, et al. Ibrutinib-associated invasive fungal diseases in patients with chronic lymphocytic leukaemia and non-Hodgkin lymphoma: An observational study. Mycoses. 2019;62(12):1140–7. 10.1111/myc.13001 31520441

[61] Varughese T, Taur Y, Cohen N, Palomba ML, Seo SK, Hohl TM, et al. Serious infections in patients receiving ibrutinib for treatment of lymphoid cancer. Clin Infect Dis. 2018;67(5):687–92. 10.1093/cid/ciy175 PMC6093991 29509845

[62] Tian Y, Ma J, Wang W, Zhang L, Xu J, Wang K, et al. Resveratrol supplement inhibited the NF-κB inflammation pathway through activating AMPKα-SIRT1 pathway in mice with fatty liver. Mol Cell Biochem. 2016;422(1–2):75–84. 10.1007/s11010-016-2807-x Epub 2016 Sep 9. 27613163

[63] Feng M, Zhang Q, Wu W, Chen L, Gu S, Ye Y, et al. Inducible Guanylate-Binding Protein 7 Facilitates Influenza A Virus Replication by Suppressing Innate Immunity via NF-κB and JAK-STAT Signaling Pathways. J Virol. 2021;95(6):e02038–20. 10.1128/JVI.-20. Print 2021 Feb 24. PMC8094947 33408175

[64] Popat R, Plesner T, Davies F, Cook G, Cook M, Elliott P, et al. A phase 2 study of SRT501 (resveratrol) with bortezomib for patients with relapsed and or refractory multiple myeloma. Br J Haematol. 2013;160(5):714–7. 10.1111/bjh.12154 23205612

[65] Ambrosone CB, Zirpoli GR, Hutson AD, McCann WE, McCann SE, Barlow WE, et al. Dietary supplement use during chemotherapy and survival outcomes of patients with breast cancer enrolled in a cooperative group clinical trial (SWOG S0221). J Clin Oncol. 2020;38(8):804–14. 10.1200/JCO.19.01203 PMC7062457 31855498

