# Supplementary figures and images for: Babesia microti-induced fulminant sepsis in an immunocompromised host: A case report and the case-specific literature review
Source: Open Life Sci. 2022 Sep 14;17(1):1200–7. doi: 10.1515/biol-2022-0448 (PMC9483830; doi:10.1515/biol-2022-0448)

# Supplementary material

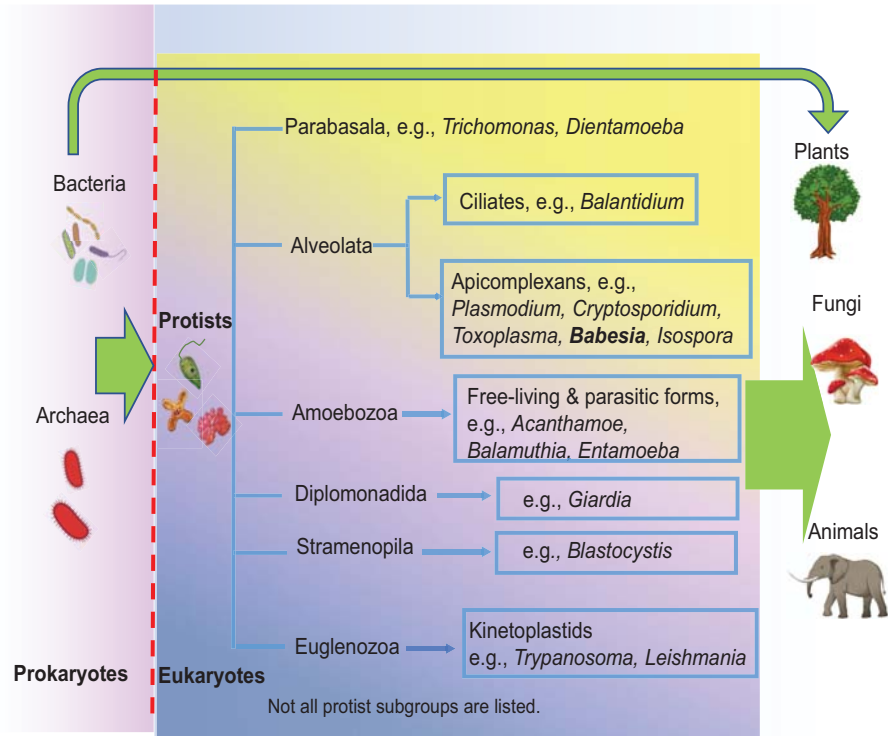

Figure S1: Apicomplexans in the evolutionary ladder.

Supplement: Supplementary Figure [file biol-2022-0448-sm.pdf]
